# Perceptions of Delta-8 THC and the Impact of a Brief Educational Video Intervention for College Students

**DOI:** 10.1007/s11414-025-09983-x

**Published:** 2025-11-19

**Authors:** Destin Rothe, Erica K. Yuen, Kathleen A. Moore, Cynthia E. Gangi, Mary Martinasek

**Affiliations:** 1 Department of Experimental Psychology, University of Oxford, Oxford, UK; 2 Department of Psychology, University of Tampa, Tampa, FL USA; 3 Department of Behavioral Health Science and Practice, University of South Florida, Tampa, FL USA; 4 Department of Health Sciences and Human Performance, University of Tampa, Tampa, FL, USA

## Abstract

Delta-8 THC is a psychoactive cannabinoid typically synthesized from hemp, with similar intoxicating effects as delta-9 THC. Surging public interest alongside the lack of federal regulation of delta-8 THC has led to an unclear legal landscape and increasing safety concerns. Educating young adults about the dangers of delta-8 THC is imperative. The current study investigated the effects of a brief educational video about delta-8 THC for college students. First, to help develop the intervention, an exploratory online survey was administered (*N* = 291) to gather information about perceptions of delta-8 THC and motivations for use. Mixed-methods analysis indicated that many students perceive delta-8 THC to have weaker (less intense, shorter-lasting) effects while being beneficial for mental and physical health. A strong motive for consumption was to enhance positive feelings, while conformity was a significantly weaker motive. These results informed the development of an educational video for students to highlight the risks of delta-8 THC and improve decision-making. Participants (*N* = 120) were randomly assigned to watch either a brief educational video about delta-8 THC or an unrelated control video about attending college. Results found that the educational video increased knowledge about delta-8 THC across all students, and lowered intentions to use delta-8 THC specifically for students who reported prior but not recent use of the substance. Perceived benefits, perceived costs, and attitudes towards legislation were not affected. Overall, results demonstrate support for the format of a brief stand-alone video intervention to increase knowledge and reduce behavioral intentions regarding delta-8 THC.

Delta-8 THC, a cannabinoid synthesized from hemp, is chemically similar to delta-9 THC,^1^ which is the main cannabinoid in marijuana.^2^ Given equivalent dosages, delta-8 THC produces similar, but overall milder, psychoactive effects compared to delta-9 THC.^3,4^ However, synthetically derived delta-8 THC, often consumed through vaping or edibles,^5,6^ has raised growing concerns among organizations such as the U.S. Food and Drug Administration (FDA) and the Centers for Disease Control and Prevention (CDC) in recent years.^7,8^ A year prior to the time of this study, between January 1, 2021, and February 28, 2022, a total of 2,362 delta-8 THC exposure incidents were reported to the National Poison Control Centers.^7^ However, the CDC noted that these reports significantly underestimate the actual number of cases.^8^ Affected individuals experienced adverse events such as paranoia, psychosis, difficulty breathing, low blood pressure, or coma, requiring evaluation in healthcare facilities or critical care units.^7–9^ Because delta-8 THC has grown in popularity only recently in the United States (US) following the passage of the 2018 Agriculture and Nutrition Improvement Act (Farm Bill), research is scarce regarding long-term consequences of use.

The 2018 Farm Bill effectively removed hemp from the list of Schedule 1 drugs (i.e., drugs with high potential for abuse and not approved for medical use), thereby exempting it from any scheduling under the Controlled Substances Act.^10,11^ This change created a federal loophole allowing for delta-8 THC synthesized from hemp to be sold legally as an unregulated product.^1,8,12^ Subsequently, most states have enacted regulations on the purchase and consumption of delta-8 THC products, ranging from essential bans (e.g., New York) to specific restrictions such as limiting sales to cannabis dispensaries (e.g., Connecticut), or introducing a legal minimum age for purchase (e.g., Kentucky).^13^ The legal landscape is constantly changing as individual states continue to put forth, consider, and pass laws regarding the regulation of delta-8 THC. At the time of this study, multiple states (e.g., Alabama, New Mexico, and more) lacked restrictions on this substance.

Because many states have not instituted any laws or policies or have enacted permissive ones,^13^ a multitude of issues have arisen. For example, due to its unclear legal status in some states, delta-8 THC products are available not only in smoke shops or CBD stores but also in convenience stores, gas stations, and online venues, where minors may be able to obtain these products.^7,14^ Additionally, the levels of delta-8 THC and other ingredients may not match the amounts listed on the product labels and can vary significantly from package to package, rendering the consumed dosage of delta-8 THC inaccurate and unpredictable.^1,8^ Some products contain larger amounts of concentrated THC than those naturally occurring in regular marijuana, resulting in effects that are more variable and potentially harmful.^7^ The US Cannabis Council has detected heavy metals as well as copper, chromium, and nickel above the US Pharmacopeia limit for inhalation in multiple samples,^6^ and the FDA has raised concerns about hygiene and involvement of potentially toxic chemicals in the production of delta-8 THC.^7^ Furthermore, many delta-8 THC products have been found to contain significantly larger delta-9 THC concentrations than the hemp-based legal federal limit of 0.3%.^6^

As concerns about delta-8 THC continue to mount, there remains a dearth of information on public knowledge, motives, and attitudes regarding delta-8 THC.^15^ Online surveys and qualitative interviews with adult^3,4^ and young adult^15^ convenience samples have found common motivators for use that include both psychological (e.g., reduce stress, anxiety, and depression) and health-related factors (e.g., reduce pain, improve sleep), as well as the ability to avoid legal risks in areas where delta-8 THC is legal but marijuana is not. Consumers also perceived delta-8 THC to be more beneficial and produce fewer side effects compared to delta-9 THC.^16^

Since young adults have the highest rate of marijuana use, with 22% of those aged 18 to 25 reporting consumption,^17^ it is important to examine this population’s perceptions and attitudes toward delta-8 THC. Recent research suggests that young adults may prefer consuming the substance in social situations versus a solitary setting and may view their experience with delta-8 THC in a positive manner.^15^ Furthermore, young adults’ social networks act as primary sources of information about such substances,^15^ which can lead to misperceptions and misjudgments in cost–benefit decision-making. Innovative and effective methods to educate young adults about the risks of using delta-8 THC are needed. Because brief video-based interventions are accessible, cost-effective, and appealing to younger audiences,^18–21^ they represent a promising avenue for influencing college students’ perceptions of delta-8 THC. Given the substance’s only recent surge in popularity, prevention and intervention research for delta-8 THC are in early stages, underscoring the need for timely educational tools to help young adults make informed decisions about use.

The purpose of the current research is twofold. The first phase of this study sought to better understand the determinants of decision-making and utilized an exploratory online survey to investigate college students’ experiences with, perceptions of, and motives to use delta-8 THC. The information gathered from the survey was subsequently used to develop an intervention video to educate college students about the nature of delta-8 THC and its potential concerns. The second phase of this study evaluated the impact of this educational video on college students’ knowledge, attitudes, and intentions regarding delta-8 THC use. It was hypothesized that the intervention video would (1) increase college students’ knowledge about delta-8 THC, (2) strengthen perceived costs associated with using the substance, (3) weaken perceived benefits associated with using the substance, (4) strengthen attitudes supporting legislation to regulate the substance, and (5) lower intentions to use. Given the recent surge in delta-8 THC's availability and the related lack of empirical research on young adults’ perceptions and intervention strategies, this report is presented as a Notes from the Field to offer preliminary insights into college students' views and the efficacy of a targeted educational video. At the time of data collection (October – December 2022 for the exploratory survey; March – April 2023 for the intervention video), delta-8 THC was unregulated in the state of Florida, where the research occurred.

## Phase 1: Exploratory Survey

### Method

#### Participants and procedures

University students were recruited via Sona Systems,^22^ a participant pool software program. Individuals were eligible to participate if they were at least 18 years old, fluent in English, and enrolled in a General Psychology course at the university where the study was conducted (a medium-sized university in West Central Florida). Approval was obtained from the university’s Institutional Review Board (IRB # 22–0660). Participants completed the study via a Qualtrics survey link. Participants read and signed an online consent form and completed qualitative and quantitative items assessing perceptions and experiences with delta-8 THC. Participants were informed that their responses would remain confidential, that participation was voluntary, and that they could skip any questions or withdraw at any time without penalty. Survey instructions included reminders to complete the study in one sitting and to avoid distractions or looking up information during participation. In the end, a debriefing statement was provided which included resources to contact if they had any mental health or substance use concerns.

#### Measures

To measure exposure to delta-8 THC, participants were first asked if they had heard of delta-8 THC (*Yes / No*) and if they had prior experience using it (*Within the past 30 days / Prior to the past 30 days / No*). Based on their responses to these two multiple-choice items, participants were presented with a series of qualitative and quantitative items. For the qualitative questions, participants were asked a series of open-ended questions about their perceptions of and experiences with delta-8 THC (see Table 1). These included items about perceived benefits and costs, reasons for non-consumption, first-use experience, and perceived use rate in peers. Participants were also asked “If an informational video were made to educate college students about delta-8 THC, what would you consider important to include?”.

**Table 1:** Open-ended survey questions for participants who had heard of Delta-8 THC (N = 291)

	History of consumption (*n* = 102)	No history of consumption (n = 189)
When you hear of Delta-8 THC, what comes to mind?	X	X
How have you consumed Delta-8 THC? Please include all the ways you have ever used Delta-8 THC	X	
Describe the circumstances of the first time you consumed Delta-8 THC. (How old were you? How did you hear about and get Delta-8 THC? Who influenced you most to try it?)	X	
What do you believe are the benefits of consuming Delta-8 THC?	X	X
What do you believe are the downsides of consuming Delta-8 THC?	X	X
In your opinion, how do the effects of Delta-8 THC compare to the effects of Delta-9 THC (regular cannabis/marijuana)?	X	X
What percentage of college students do you think have tried Delta-8 THC?	X	X
For what reasons have you never consumed Delta-8 THC?		X
If an informational video were made to educate college students about Delta-8 THC, what would you consider important to include?	X	X

For the quantitative questions, students who reported prior use of delta-8-THC completed a 25-item self-report instrument adapted from the Marijuana Motives Measure (MMM),^23^ with items rated on a 5-point Likert scale (1 = *Almost Never/Never* to 5 = *Almost Always/Always*). This original measure demonstrates strong concurrent validity and internal consistency across five factors: 1) enhancement (use to induce positive emotion); 2) coping (use to reduce negative emotion); 3) sociability (use to improve social experiences); 4) conformity (use to fit in); and 5) expansion (use to increase awareness and insight), with internal consistency ranging from α = 0.70 to 0.93.^23,24^ Adapting established substance use measures for related substances is common in substance use research, and is often necessary for emerging substances for which validated measures do not yet exist. ^25,26^ Consistent with prior work which adapted the MMM for delta-8 THC-related research,^27^ the MMM was modified slightly for the present study by replacing the term “marijuana” with “delta-8-THC.” Internal consistency in the current sample was maintained and ranged from α = 0.86 to 0.92. Participants with prior delta-8-THC use also responded to two additional items, adapted from Kruger and Kruger,^16^ which assessed perceived differences between delta-8 THC and delta-9 THC in terms of strength (1 = *Delta-8 THC is much more intense* to 5 = *Delta-9 THC is much more intense*) and duration (1 = *Delta-8 THC lasts a lot longer* to 5 = *Delta-9 THC lasts a lot longer*).

#### Analysis

Data analysis was conducted using IBM SPSS Statistics V.29. To assess differences in the strength of various motives for consuming delta-8 THC, a within-subjects ANOVA was conducted across the five factors in the adapted Motives Measure. Secondly, descriptive statistics were used to examine perceived effects of delta-8 THC compared to delta-9 THC. Third, a qualitative analysis was conducted on each open-ended survey item to identify common themes related to the usage, perceived benefits, perceived costs, and effects of delta-8 THC. An inductive content analysis approach^28^ was used, with each response coded line by line to identify and quantify common categories, such as benefits to mental health and potential harms to physical health. Using Microsoft Excel, two trained research assistants independently coded the responses for each item. A third research assistant reviewed discrepancies and facilitated resolution through collaborative discussion with the original coders. All coding activities were supervised by a principal investigator (second author). Subsequent coding passes by the first and second authors reviewed the data for clarity, accuracy, and non-redundancy, merging codes into broader categories when appropriate. Discrepancies were resolved through discussion and consensus.

### Results

Participant (*n* = 291) ages ranged from 18 to 23 years (*M* = 19.1, *SD* = 1.2). The sample consisted mostly of freshmen (47%) and sophomores (30%) with some juniors (13%) and seniors (10%). The majority of participants identified as female (69%). Most were White (87%), with others identifying as African American (4%), Multiracial (4%), Asian (2%), Native American (< 1%), and Other (3%). Regarding familiarity and use of delta-8 THC, most participants (71%; *n* = 207) had heard of delta-8 THC prior to this study. However, only 35% of participants (*n* = 102) reported prior consumption of delta-8 THC, either within the past 30 days (*n* = 41), or prior to the past 30 days (*n* = 61).

#### Motives

For participants with a history of consumption (*n* = 102), a within-subjects ANOVA demonstrated significant differences amongst the motives for using delta-8 THC, *F*(4,384) = 75.18, *p* < 0.001, η_p_^2^ = 0.439. Post-hoc pairwise comparisons with Bonferroni adjustments found that the enhancement motive (i.e., because it is fun and produces positive feelings) was significantly higher (*M* = 15.8, *SD* = 5.4), while the conformity motive (i.e., following social norms to fit in) was significantly lower (*M* = 7.3, *SD* = 3.3) than the other motives, all *ps* < 0.001. Means and standard deviations of the adapted Motives Measure are displayed in Table 2.

**Table 2:** Mean scores and standard deviations for the five factors of the motives measure

Motive	M	SD
Enhancement	15.75*	5.37
Coping	12.34	5.11
Expansion	11.56	5.39
Social	11.39	5.56
Conformity	7.30*	3.30

^*^significantly different from other motives at *p* < 0.001 via Bonferroni-corrected pairwise comparisons

*Note.* Participants with a history of consumption completed this measure (*n* = 102)

#### Comparison of perceived effects

Overall, participants with a history of consumption tended to perceive the effects of delta-8 THC as less intense compared to delta-9 THC (72%) (see Table 3). Additionally, many participants (50%) reported that the effects of delta-8 THC were shorter-lasting compared to delta-9 THC, while only 13% of participants reported delta-8 THC to be longer lasting.

**Table 3:** Consumers’ perceived strength of Delta-8 THC compared to Delta-9 THC (i.e., regular marijuana)

	*n*	%
Intensity		
Delta-8 THC is much more intense	4	3.9
Delta-8 THC is somewhat more intense	4	3.9
About the same	5	4.9
Delta-9 THC is somewhat more intense	38	37.3
Delta-9 THC is much more intense	35	34.3
Do not know	16	15.7
Duration		
Delta-8 THC lasts a lot longer	2	2.0
Delta-8 THC lasts a little longer	11	10.8
About the same	18	17.6
Delta-9 THC lasts a little longer	26	25.5
Delta-9 THC lasts a lot longer	25	24.5
Do not know	20	19.6

Participants with a history of consumption completed this measure (*n* = 102)

#### Content analysis

Several common categories were identified in the areas of 1) usage; 2) perceived benefits; 3) perceived costs; and 4) comparisons to marijuana. Most participants (74%) reported peers as their main influence for their first delta-8 THC experience. Many reported being 18 years or older (55%) at the time of their first experience. Users primarily consumed delta-8 THC through inhalation (86%) and edibles (48%), with vaporizers being a common method of inhalation (67%). Most participants believed delta-8 THC use was widespread across the college student population. For those without a history of consumption, commonly cited reasons for abstaining included a lack of interest (74%), concerns about health risks or potential side effects (35%), and general avoidance of recreational drug use (20%).

Most participants who knew of delta-8 THC reported at least one perceived benefit. Commonly cited benefits included: feelings of enjoyment (55%), such as the relaxing and calming effects on body and mind; mental health benefits (44%), such as anxiety reduction and stress relief; and benefits to physical health (29%), such as improved sleep and pain management. Commonly cited perceived costs included: potential harm to physical health (50%), for example, lung damage, slower/worse brain functioning, and memory loss; mental health concerns (28%), such as low motivation or productivity, anxiogenic effects, and tiredness; and the potential for addiction and dependency (24%).

Many participants (55%) indicated perceived differences compared to marijuana, with 32% believing delta-8 THC to have weaker effects, while few perceived delta-8 THC as stronger. Some participants also indicated their belief that delta-8 THC was safer and less risky in terms of side effects. Only 14% of participants expressed concerns about the ingredients or production of delta-8 THC.

#### Suggestion for an educational video

In response to this prompt, participants gave a variety of suggestions for an educational video, with many emphasizing the need to cover “both the positive and negative effects it has so people know fully what they are doing and how it is impacting their body.” Other responses highlighted the importance of explaining “how it compares to regular weed.” Additionally, several participants expressed interest in understanding the legal status of delta-8 THC and receiving guidance on how to use it safely.

### Discussion

Results from the exploratory survey suggest that experiencing positive feelings is a stronger motivator for delta-8 THC use in college students while peer pressure may be a weaker motivator comparatively. Perceived benefits of delta-8 THC include increased enjoyment and relaxation, reduced anxiety and stress, and improved sleep and pain management. These results are consistent with initial research on motivating factors in delta-8 THC use.^5,6,15^ In addition, the results found that perceived costs of consumption largely centered on potential harm to physical and mental health, as well as the potential for addiction and dependency.

Only a minority of students exhibited knowledge about safety concerns specific to delta-8 THC products. The majority of participants believed delta-8 THC to be weaker and shorter-lasting than delta-9 THC, with some noting that delta-8 THC is more calming, produces less psychoactive effects, and helps achieve a more functional high. These findings, which are in line with the results of Kruger and Kruger,^16^ suggest that many college students may view delta-8 THC products as a safer alternative compared to delta-9 THC marijuana products. Overall, results suggest a significant knowledge gap and misinformation concerning the potential dangers of delta-8 THC. This gap includes lack of awareness about the potential contaminants, unregulated production processes, and overall health risks associated with delta-8 THC products.

## Phase 2: Video Intervention

Results from Phase 1 were used to develop a short educational video to inform college students about the risks of using delta-8 THC. The design of the video was guided by the limited but promising evidence on brief web and video-based interventions for substance use, which suggests that short length, relatable wording, personal narratives, and neutral/balanced content (e.g., no “fear mongering”) may increase the responsiveness to a video intervention designed for young adults.^29–33^ Furthermore, given the research support for motivational interviewing^34^ in substance use risk reduction programs for college students,^29^ several of its principles were incorporated into the video, including the exploration of both pros and cons for using versus not using the substance, with an emphasis on self-determination and personal responsibility for making the best decision for oneself. The video devoted slightly more time to the drawbacks of using the substance as opposed to benefits, to subtly tilt in favor of not using the substance while still appearing to be neutral. Of the benefits mentioned, the video focused on weaker motivators (e.g., fitting in, reducing peer pressure) but made minimal mention of motives that the exploratory survey found to be more compelling to college students (e.g., enjoying the high). The 4-min video contained five sections:

1. A voice-over providing an overview of delta-8 THC and its common effects, difference to marijuana products, and unregulated status.
2. “Positive” testimonials from student actors (2 males, 2 females) on how delta-8 THC has helped them to fit in socially, sleep, and avoid legal trouble. Common circumstances of first exposure (e.g., social situation with other students, purchasing from a store) and colloquialisms (e.g., “marijuana lite”) observed in the responses of the exploratory survey were incorporated into the testimonials.
3. A voice-over section on safety concerns associated with delta-8 THC due to its unregulated status (harmful chemicals, inaccurate labeling, hospitalizations, deceptive marketing toward youth) and how it had not been declared safe by the FDA.
4. “Negative” testimonials from different student actors (2 males, 2 females), such as immediate and day-after side effects (e.g., anxiety, grogginess, difficulty in functioning), its addiction potential, concerns about unsafe materials inside the product, dislike of sneaky marketing techniques, and preference for alternative ways of coping with stress (e.g., exercise, meditation).
5. A voice-over summary of how college students report both benefits and disadvantages to using delta-8-THC, and encouragement to the viewer to make their own informed decision.

### Method

#### Participants and procedures

University students were recruited via Sona Systems. Individuals were eligible to participate if they were at least 18 years old, fluent in English, and enrolled in a General Psychology course at the university where the study was conducted at a medium-sized university in West Central Florida. Approval was obtained from the university’s IRB (IRB # 23–003). All participants completed the study online via Qualtrics. Participants were informed that the study examined college students’ responses to informational videos, without specifying the focus on delta-8 THC. After providing online consent, participants were instructed to complete the study in one sitting on a computer in a quiet location, avoiding distractions. Participants were randomly assigned to one of two conditions via Qualtrics’s randomizer function, set to ensure even distribution. The experimental group watched the 4-min educational video intervention about delta-8 THC. The control group watched a similarly structured 4-min video about the pros and cons of attending college. Both videos used the same actors and format (images, narration, and student testimonials) and emphasized personal autonomy and informed decision-making.

After watching their assigned video, participants were asked to type the topic of their video into a textbox. Control group participants unfamiliar with delta-8 THC received a brief definition. All participants then completed questionnaires assessing knowledge, perceived benefits, perceived costs, attitudes towards legislation, and intentions to use. Next, participants were asked to describe their reaction to the video, which the researchers monitored to ensure the intervention did not unintentionally promote delta-8 THC use. Participants then read a debriefing statement informing them of the true nature of the study and were provided with mental health and substance use resources. Finally, control group participants had the option of viewing the intervention video during debriefing.

#### Measures

Participants completed several instruments assessing knowledge, beliefs, behavioral intentions, and attitudes related to delta-8 THC. Knowledge was assessed with a 15-item true–false quiz. The measure was developed by the research team based on (1) key themes identified in the exploratory survey, (2) FDA and CDC health communications, (3) information from a community-based public health advocate, and (4) input from four behavioral health faculty experts. The development process emphasized clarity, relevance to real-world policy and health concerns, and alignment with the video content. Items were reviewed for content coverage and face validity to ensure appropriateness for a college student population. Content included information about legal status, availability, side effects, and health concerns.

Participants’ perceived benefits and drawbacks of delta-8 THC use were assessed using an adapted version of the Decisional Balance Scale, which has been widely adapted for various behaviors and substances, including tobacco,^35^ alcohol,^36^ and marijuana.^37^ Development of this measure is grounded in the Transtheoretical Model of Behavior Change, which asserts that individuals’ weighing of pros and cons of a behavior (e.g., substance use) shapes their decision to adopt or refrain from that behavior, and this framework applies broadly across a diverse range of health behaviors.^38^ In a 24-item version developed for marijuana use, confirmatory factor analysis supported a two-factor structure (perceived benefits and perceived costs) of motivational factors in young adults, with strong internal consistency (benefits α = 0.91; costs α = 0.95).^37^ This measure has participants rate the importance of each benefit and cost in their decision to use the substance (1 = *Not at all important* to 5 = *Very important*). For the current study, the Decision Balance Scale was adapted by replacing references to “marijuana” with “Delta-8-THC.” In addition, the item "It’s illegal, and I could get caught" was revised to "It is not allowed on campus, and I could get caught." Internal consistency was maintained in the current sample (benefits α = 0.92; costs α = 0.95).

Behavioral intentions were assessed on a 3-item measure adapted from Elliot et al.^37^ (α = 0.93), which asked participants how likely they were to use marijuana within the next week, next month, and at any point in the future (1 = *Definitely will not* to 6 = *Definitely will*). For the current study, the items were modified to ask about delta-8 THC instead of marijuana, with internal consistency maintained (α = 0.91 for this study). This measure of intention is consistent with the Theory of Planned Behavior (TPB) ^39^ and the Theory of Reasoned Action (TRA),^40^ both of which assert a predictive link between substance use intentions and actual behavior. Intention to use has been shown to be a strong predictor of actual behavior in substance use research and is commonly assessed in substance use studies.^41,42^

Attitudes toward legislation of delta-8 THC were assessed using a 12-item instrument developed for the current study. Given the absence of established measures assessing policy views on delta-8 THC, the researcher team developed this instrument to evaluate participants’ opinions on legal regulation of the substance. The development of this instrument was informed by behavioral theory (e.g., TPB, TRA, Health Belief Model^43^), recognizing that students’ legislative attitudes may reflect their perceptions of delta-8 THC’s risks, benefits, and social acceptability, and thus function as an indicator of future usage. Item development was guided by themes identified in the exploratory survey (e.g., health-related concerns, methods of access), FDA and CDC health communications, information from a community-based public health advocate, and review by four behavioral health faculty experts for face and content validity. Items were rated on a 5-point Likert scale (1 = *Strongly disagree* to 5 = *Strongly agree*) for statements such as “The recreational use of delta-8 THC should be legal” and “Legal regulations should require delta-8 THC products to be kept behind the counter at stores.” Three items were reverse-coded, with higher total scores indicating stronger support for regulation (α = 0.77 for this study). An additional open-ended item asked participants what they considered the most appropriate legal minimum age for delta-8 THC use.

#### Analysis

IBM SPSS Statistics V.29 was utilized for data analysis. Given the intent to independently interpret the dependent variables as distinct constructs, alongside concerns about balancing Type I and Type II error, particularly because this is a new area of research, the researchers elected a priori to use Bonferroni-adjusted post-hoc tests to control for family-wise error within a given test, but not adjust alpha to control for experiment-wise error. For the main analyses, a series of independent samples *t*-tests were conducted to assess whether viewing the intervention video versus control video led to differential effects on the dependent variables. For the secondary analyses, two-factor ANOVAs were conducted to explore whether outcomes may be influenced by prior experience with Delta-8 THC. For any significant interaction, the main effects were not interpreted.

### Results

A total of 132 General Psychology students completed the study. Twelve participants were removed from the analysis for failing to pass the validity check which asked for a sentence describing the video that they watched. This left a sample size of 120 participants (experimental *n* = 58; control *n* = 62). Participant ages ranged from 18 to 39 years with a mean age of 19.8 (*SD* = 2.2). Most participants were sophomores (56%), with freshmen (21%), juniors (14%), and seniors (9%) rounding out the sample. The majority of participants identified as female (62%) and White (91%) with other participants identified as African American (3%), Asian (3%), Multiracial (3%), and Other (1%). Eight percent of the participants identified as Hispanic. Most participants (78%; *n* = 94) had heard of delta-8-THC prior to the study. The majority of participants (58%; *n* = 70) reported they had never consumed delta-8 THC, with 11% (*n* = 13) consuming within the past 30 days and 31% (*n* = 37) consuming more than 30 days ago.

#### Main analyses: Effects of intervention on knowledge, perceived costs/benefits, attitudes toward legislation, and intentions

Independent samples *t*-tests found that participants who watched the intervention video achieved significantly higher knowledge quiz scores about delta-8 THC (*M* = 12.5, *SD* = 2.2) compared to participants who watched the control video (*M* = 11.1, *SD* = 2.1), *t*(118) = −3.64, *p* < 0.001, *d* = 0.67. No significant results were found for perceived benefits, perceived costs, attitudes towards legislation, or intentions to use delta-8 THC (see Table 4). Of note, when participants described their reaction to their assigned video, no responses indicated that the video increased their interest in using delta-8 THC.

**Table 4:** Means, standard deviations, and results for main outcomes (N = 120)

	Video Intervention Condition		Control Condition		*t*(118)	*p*
	*M*	*SD*	*M*	*SD*		
Knowledge quiz	12.5	2.2	11.1	2.1	3.23	< 0.001*
Perceived benefits	20.2	7.4	20.5	8.8	0.23	0.817
Perceived costs	59.8	14.7	59.7	14.6	−0.50	0.960
Attitudes toward legislation	44.9	5.8	44.6	6.9	0.01	0.778
Recommended min legal age	19.3	1.7	19.6	1.9	0.81^	0.422
Intentions to use	5.8	4.1	5.8	4.2	0.15	0.960

^*^significant at α < .05

^ *df* = 116

#### Secondary analyses: Prior consumption of delta-8 THC as a moderator

Two-factor ANOVAs found a significant interaction between condition and consumption history on intentions to consume delta-8 THC in the future (see Table 5). Bonferroni-corrected pairwise comparisons found that participants whose most recent delta-8 THC consumption was *prior to* the past 30 days demonstrated significantly lower intentions to consume in the future (*p* = 0.004) if they had watched the educational video (*M* = 5.0; *SD* = 2.4) compared to the control video (*M* = 8.1; *SD* = 3.9). For participants who had *never* consumed or had consumed *within* the past 30 days, no significant differences for intentions to consume were found between conditions. No other significant interactions were found for prior consumption as an influencing factor.

**Table 5:** Two-way ANOVA results for condition and consumption history (N = 120)

		*df*	*F*	*p*	η_p_^2^
Knowledge quiz					
Condition		1, 114	6.67	0.011*	.06
History		2, 114	1.59	0.209	.03
Condition x History		2, 114	1.75	0.179	.03
Perceived benefits					
Condition		1, 114	0.56	0.458	.01
History		2, 114	5.41	0.006*	.09
Condition x History		2, 114	0.85	0.430	.02
Perceived costs					
Condition		1, 114	0.00	0.994	.00
History		2, 114	8.47	< 0.001*	.13
Condition x History		2, 114	0.50	0.606	.01
Intentions to use					
Condition		1, 114	0.00	0.988	.00
History		2, 114	30.50	< 0.001*	.35
Condition x History		2, 114	4.96	0.009*	.08
Attitudes toward legislation					
Condition		1, 114	0.37	0.546	.00
History		2, 114	8.43	< 0.001*	.13
Condition x History		2, 114	0.66	0.519	.01
Recommended minimum legal age					
Condition		1, 112	0.51	0.478	.01
History		2, 112	3.77	0.026*	.06
Condition x History		2, 112	0.13	0.883	.00

^*^significant at α < .05

Main effects for consumption history, with Bonferroni post-hoc comparisons, found that those who had never consumed delta-8 THC reported significantly greater perceived costs of usage compared to those who had consumed within (*p* = 0.015) and prior to (*p* < 0.001) the past 30 days, and significantly lower perceived benefits compared to those who had consumed within the past 30 days (*p* = 0.009). Main effects also found that those who had never consumed held significantly stronger beliefs that government should pass legislation to regulate delta-8 THC compared to those who had consumed within (*p* = 0.016) and prior to (*p* = 0.001) the past 30 days. Non-consumers also recommended an average minimum legal age (*M* = 19.9; *SD* = 1.9) that was significantly higher than the age recommended by those who had consumed prior to (*M* = 18.9; *SD* = 1.7) the past 30 days (*p* = 0.023).

### Discussion

The findings support the hypothesis that the brief video intervention would increase college students’ knowledge about delta-8 THC. Furthermore, the video reduced intentions to consume delta-8 THC, although this finding was only significant for students who had a past (but not current) history of consuming the substance. Contrary to the hypotheses, the video did not seem to affect perceived costs and benefits or attitudes about government regulation. Findings from this study are partially in line with results from Ajumobi and colleagues,^33^ which demonstrated that a brief educational video could affect behavioral intentions regarding substance use. Unlike their study, however, the current study did not observe changes in harm beliefs.

For college students who have recently used delta-8 THC, watching a short 4-min video may not have strong enough impact to alter attitudes about the recreational use of THC products, especially if they view their experience with delta-8 THC in a positive manner. Risk/benefit perceptions of delta-8 THC in users are complex and influenced by a variety of social, political, and legal factors.^16^ It is possible that a longer or more immersive intervention (e.g., a series of short videos or interactive multimedia/gamified activities) could be more impactful for this population, although more intensive formats may also increase the risk of inattentiveness and non-completion. In addition, student testimonials in the current video intervention were short, ranging from 10 to 15 s per testimonial, but longer testimonials could potentially foster a stronger emotional connection with the testimonial givers, thus more powerfully impacting attitudes and behavioral intentions regarding delta-8 THC. While attitudes about risks versus benefits appeared unaffected in the current study, the reductions in intentions to consume may reflect increased perceived behavioral control over delta-8 THC consumption and the perception that abstaining is socially acceptable—two constructs central to TPB, which asserts that intentions and behaviors are collectively shaped by perceived control, subjective norms, and attitudes.^39^

Results from the current study also found that attitudes about delta-8-THC in college students who had never consumed the substance differ compared to those who have consumed. Those without history endorsed stronger perceived costs, weaker perceived benefits, stronger government regulation of the substance, and a higher minimum age. These views about delta-8 THC may be reflective of their overall stance toward substance use in general, including reasons for not partaking.

## General Discussion

Given the recent surge in delta-8 THC's availability and the related lack of empirical research on its perceptions and interventions among young adults, this report offers preliminary insights into college students' views and the efficacy of a targeted educational video. Findings from the Phase 1 survey suggested that many students may be unaware of its potential dangers and view delta-8 THC as a safer alternative to marijuana. Furthermore, pleasure-seeking emerged as a stronger motive than peer influence. These insights informed the development of the Phase 2 brief educational video which increased student knowledge about Delta-8 THC and reduced intentions to use it among those with a past, although not recent, history of consumption. These results align with the growing body of research demonstrating that video-based interventions may be a helpful option for behavior change.^32,33^ Since video-based substance use interventions have the potential advantage of being cost-effective and easily accessible^19^, there is great potential to utilize this modality for large-scale educational outreach. Further research is needed to explore impactful community-based digital intervention strategies.

### Limitations

The results of this study must be interpreted within the context of the study’s limitations. First, participants in both parts of the study completed their online tasks independently in a location of their choosing as opposed to in a controlled laboratory setting. Although participants in the intervention video analysis passed a basic validity check, it is unknown to what extent they were attentive throughout the entire video. Secondly, because delta-8 THC research is in its infancy, no psychometrically validated measures exist for this substance. Therefore, measures were adapted from established substance use instruments^23,37,38^, and new instruments developed based on theoretical frameworks,^39,40,43^ exploratory survey findings, and expert review. Although factor analysis was not feasible, the measures used in the current study demonstrated adequate to excellent internal consistency in the sample, and the items reflected themes identified in the Phase 1 survey. Together, these elements provide preliminary psychometric support, justifying their use in this exploratory investigation of an emerging substance, particularly given growing public health concerns and the need for scalable interventions. Third, it is possible that the true/false format of the knowledge quiz affected the results, as participants had a 50% chance of guessing a particular response correctly. A better option may have been to give participants the option of answering “Unsure” to each question, which could result in quiz scores that better represent knowledge of Delta-8 THC. Fourth, only short-term impact of the video intervention was assessed, as the dependent variables were measured immediately after watching the assigned video. Future research should also investigate potential long-term impacts of a video intervention, after days or weeks have passed, giving opportunity for participants to reflect further upon the information presented to them.

There are also limits to external validity, as both samples consisted largely of white, female underclassmen at the same university, all enrolled in General Psychology. Results could have been different with more upperclassmen participants, especially since exposure to Delta-8 THC toward the beginning of the college experience may be particularly impactful.^44^ In addition, students enrolled in a General Psychology course, whether for their major or general education purposes, may have different attitudes toward substance use compared to students in the general university population. It should also be noted that this research study occurred in a state (Florida) where marijuana is illegal, and Delta-8 THC was unregulated at the time of data collection. Participants were also located on a metropolitan campus surrounded by multiple smoke shops and gas stations legally selling Delta-8 THC products. Hence, the awareness and popularity of hemp products might have been higher for these study participants compared to students at other colleges and universities. In states where marijuana is legal, or where Delta-8 THC is illegal, the demand for and awareness of hemp products such as Delta-8 THC may be lower. Beginning July 2023, Florida put into effect a law declaring age 21 the minimum legal age to purchase Delta-8 THC products,^45^ which is likely to have cascading effects on the attitudes and behaviors of college students residing in this state.

## Implications for Behavioral Health

With the recent surge of public interest in Delta-8 THC alongside burgeoning safety concerns, it is imperative for health professionals, educators, and lawmakers to better understand the attitudes, intentions, and behaviors in adolescents and young adults related to this substance. This information is essential in the consideration of appropriate regulations and laws for psychoactive hemp products, as well as to inform the development of innovative and effective prevention/intervention programs. With the rising popularity of smartphone use and video-based social media (e.g., YouTube, Instagram, and TikTok) over the past two decades,^18^ scalable video interventions may be the next wave of the future. This medium has become a powerful means of communication, especially when targeting a younger audience.

Results from the current study suggest that the format of a brief stand-alone video intervention has the potential to increase knowledge and reduce behavioral intentions in college students regarding Delta-8 THC. Furthermore, a brief video intervention may have differential effects based on the specific characteristics of the viewer, such as history of consumption. Addressing not only attitudes toward use but also perceived control over one’s consumption and the influence of social norms around abstaining may enhance the effectiveness of these interventions. In general, video interventions should be tailored to the specific target population, seeking to connect with the viewers while incorporating evidence-based strategies and information about the target’s motives, attitudes, and behaviors. In developing an educational intervention, the strategy of encouraging personal autonomy in decision-making also requires careful consideration to prevent accidental promotion of the substance. Future research should continue to explore the development and efficacy of technology-based, widely accessible prevention/intervention methods in at-risk groups. Impactful and innovative dissemination strategies (e.g., via particular social media sites, or video posts from popular social influencers) should also be investigated.

## Data Availability

Data sets generated during the current study are available from the corresponding author on reasonable request.
